# Peripheral non‐invasive focused ultrasound alleviates constipation by reducing gastrointestinal inflammation

**DOI:** 10.1113/EP093281

**Published:** 2026-09-26

**Authors:** Dia Shah, Kainat Akhtar, Dev Dwivedi, Amal Siddiqui, Griffin Haggas, Nicole Forman, Tatiana Gervase, Yifan Kao, John Chen, Julia Brzac, Eric Molho, Damian Shin

**Affiliations:** ^1^ Department of Neuroscience and Experimental Therapeutics Albany Medical College Albany New York USA; ^2^ Department of Neurology Albany Medical Center Albany New York USA; ^3^ Department of Anatomical Sciences and Neurobiology University of Louisville Louisville Kentucky USA

**Keywords:** cytokine, GI inflammation, gut motility, loperamide, non‐invasive therapy, rat

## Abstract

Constipation affects approximately 20% of the US population, with diverse treatment options available due to the unclear onset and causes of the condition. Recently, focused ultrasound (FUS) has been proposed as a novel, non‐invasive peripheral neuromodulatory treatment that may reduce this inflammation. We utilized a loperamide‐induced constipation rat model, which is a widely used model to study constipation and potential therapeutic interventions. This study investigated the effects of FUS treatment on colonic inflammation and motility. We report that loperamide treatment did not alter colon morphology, but upregulated interleukin (IL)‐6, IL‐10, IL‐1ra and several other cytokines. FUS treatment notably reduced the expression of IL‐2, IL‐4, IL‐17 and other markers of inflammation. Additionally, FUS therapy also reduced the expression of chemokines, such as CXC3CL‐1 and CXCL‐7, and acute phase proteins, including vascular endothelial growth factor and intercellular adhesion molecule 1 in loperamide–FUS‐treated rats. One week post‐treatment, cytokine and chemokine profiles showed a sustained decrease of inflammation in the distal colon, with FUS treatment continuing to reduce several inflammatory mediators. These results demonstrate non‐invasive FUS markedly reduced the expression of pro‐inflammatory cytokines, chemokines and acute phase proteins in both the proximal and distal colon of loperamide‐treated rats and can plausibly serve as a therapeutic for chronic constipation and other inflammatory gastrointestinal disorders.

## Abstract (highlights)

- **What is the central question of this study?**
  Does peripheral non‐invasive focused ultrasound targeted to the celiac plexus improve constipation in a rat model?
- **What is the main finding and its importance?**
  Peripheral non‐invasive focused ultrasound targeted to the celiac plexus in a rat model of constipation increased faecal pellet numbers coinciding with altered inflammatory cytokine expression in the colon, but did not alter colon morphology.

## 1 INTRODUCTION

Up to 20% of the US general population experiences constipation, resulting in 2.5 million medical visits annually (Vazquez Roque & Bouras, 2015). Constipation is defined as less than three stool movements per week and can comprise various symptoms including stool hardening, abdominal discomfort and bloating (Jani & Marsicano, 2018). The Rome IV Criteria are commonly used in clinical trials for constipation; to qualify, patients meet two of the eight criteria, including straining during defecation, stool hardness, incomplete evacuation, obstruction and insufficient criteria to diagnose irritable bowel syndrome (Jani & Marsicano, 2018; Vazquez Roque & Bouras, 2015). The pathogenesis of constipation is highly associated with low‐grade inflammation and increased permeability of the intestinal lumen (Ju et al., 2020; Kim et al., 2021). Recurring constipation is associated with chronic illnesses found in older patients such as anorectal and colorectal diseases (Akhtar et al., 2021), metabolic diseases (Wei et al., 2023) and neurodegenerative diseases, such as Parkinson's disease (Mozaffari et al., 2020). Although the aetiology of constipation is not fully elucidated, it is strongly associated with numerous factors including age, diet, lifestyle, medications, and a history of anxiety, depression or other gastrointestinal (GI) diseases and/or disorders (Jani & Marsicano, 2018; Nelson et al., 2017; Portalatin & Winstead, 2012; Schmidt & Santos, 2014).

Current treatments for constipation focus on diet changes, increased activities of daily living, along with pharmaceutical and/or surgical interventions (Nelson et al., 2017; Wegh et al., 2022). Pharmaceutical interventions, such as laxatives, are commonly used but potentiate side effects such as dehydration, defective absorption of essential nutrients, erosive properties that diminish gut‐barrier integrity (Portalatin & Winstead, 2012; Wegh et al., 2022), and in some cases, increased intestinal dysfunction due to lumen erosion (Mozaffari et al., 2020). Furthermore, laxatives alone are estimated to cost $821 million annually in the United States (Portalatin & Winstead, 2012). When untreated, chronic constipation can lead to bowel obstructions, requiring invasive surgical interventions, including cecostomy, colectomy, and recently, implantable nerve stimulation devices (Pfeifer, 2015). Unfortunately, the latter can present with a lack or loss of benefit, pain or dysesthesia, and device complications (Bielefeldt, 2016). This aligns with other modalities of treatment as long‐term use of laxatives protracts pain (Paré & Fedorak, 2014) while surgical interventions are comorbid with aforementioned adverse effects, further diminishing patients’ quality of life (Sharma et al., 2020).

Although the pathogenesis of constipation remains incompletely understood, dysregulation of the enteric nervous system (ENS) may play a critical role in its persistence and recurrence. The GI tract within the ENS is inherently complex, containing both neural and immune components that interact to modulate motility and inflammation. In cases of constipation, there is often a dysregulation in this pathway with decreased levels of key neurotransmitters such as acetylcholine (ACh) and serotonin, both of which play critical roles in gut motility and peristalsis (Kim et al., 2018). In addition, an increase in pro‐inflammatory cytokines and markers has been observed, which can exacerbate dysfunction in the gut, contributing to motility disorders (Ju et al., 2020; Kim et al., 2018, 2021; Liu & Zhi, 2021). Recent studies highlighted the significant role of the neuroimmune pathway in the regulation of GI function, making it a promising target for the treatment of constipation (Ju et al., 2020; Kim et al., 2018; Liu & Zhi, 2021). Traditional treatments primarily address symptoms rather than the underlying mechanisms driving constipation; in contrast, a neuro‐immune targeting strategy considers the holistic interactions between the central nervous system and ENS (Tansey et al., 2022). Targeting the neuro‐immune axis offers a novel and potentially more effective approach for treating constipation.

Neuromodulation represents an emerging, minimally invasive approach to managing chronic constipation, particularly in cases associated with neuromuscular dysfunction (Jani & Marsicano, 2018; Jost, 2010; Portalatin & Winstead, 2012; Vazquez Roque & Bouras, 2015). Techniques such as vagus nerve (VN) stimulation (Payne et al., 2019) and peripheral focus ultrasound (FUS) have demonstrated the potential to enhance GI motility by directly influencing the neural pathways involved in GI homeostasis (Cotero et al., 2020). Using non‐invasive neuromodulation to minimize inflammation and restore homeostasis in the ENS may serve as a novel approach for treating constipation (Kelly et al., 2022; Pavlov & Tracey, 2015; Payne et al., 2019; Powley et al., 2019). Our lab was the first to apply non‐invasive focused ultrasound to the celiac plexus, which receives parasympathetic fibres from the VN, demonstrating its efficacy to improve GI barrier integrity in a rat model of inflammatory bowel disease (Akhtar et al., 2021). The bidirectional communication between the nervous and immune systems is particularly relevant in constipation, as impaired motility and inflammation in the GI can interfere with neural signalling.

In this study, we employed an established loperamide‐induced model of constipation (Ayari et al., 2025; Kim et al., 2018; Liu & Zhi, 2021; Wintola et al., 2010), which targets μ‐opioid receptors in the myenteric and submucosal plexuses of the GI tract (Sobczak et al., 2014). Loperamide's suppression of motility in the myenteric plexus, coupled with its impact on mucosal secretion in the submucosal plexus, effectively impairs GI function (Burleigh, 1988; Wang et al., 2023). Additionally, its inability to cross the blood–brain barrier ensures that its effects remain localized to the GI tract, making it an ideal model for evaluating the therapeutic potential of FUS neuromodulation. Here, we investigate the effects of FUS treatment on the celiac plexus on colonic inflammation, motility and morphology in loperamide‐injected rats to assess whether this neuromodulatory approach can elicit an anti‐inflammatory signal to the GI to alleviate constipation.

## 2 METHODS

### 2.1 Ethical approval

The investigators understand the ethical principles under which *Experimental Physiology* operates. Our work complies fully with the animal ethics policy of the journal. This study was conducted under an approved animal care and use protocol in accordance with the National Institutes of Health and Albany Medical College's Institutional Animal Care and Use Committee (IACUC) guidelines (no. 24‐09001). Male Sprague–Dawley rats aged 7–8 weeks old (225–250 g) were purchased from Taconic Biosciences (Germantown, NY, USA). Animals were individually housed in home cages with unrestricted access to food and water, and procedures were performed during the light phase (07.00–18.000 h) of the light–dark cycle.

### 2.2 Loperamide‐induced model of constipation

Loperamide is a peripherally acting μ‐opioid receptor agonist which impairs excitatory motor neurons in the myenteric plexus and secretomotor activity within the submucosal plexus, contributing to constipation (Sobczak et al., 2014; Wang et al., 2023). These neuronal effects, which lead to stool obstruction, are coupled with downstream alterations in the GI immune microenvironment, including increased infiltration of immune cells and pro‐inflammatory cytokine production (Jani & Marsicano, 2018). This unique overlap between ENS dysfunction and immune activation makes this model well‐suited for probing neuroimmune mechanisms in constipation and identifying FUS as a therapeutic intervention (Kim et al., 2018; Sobczak et al., 2014; Wang et al., 2023; Zhang et al., 2021). A stock solution containing 0.5% Tween 20 and 0.5 mg/mL loperamide HCl in double‐distilled water was prepared fresh daily; control animals were injected with a 0.5% Tween 20 in 0.09% NaCl solution. Here, animals were injected with loperamide or saline at 4 mg/kg body weight twice daily for three consecutive days subcutaneously using the following equation:

$$\mathrm{Volume}\;\left(\mathrm{mL}\right)=\frac{\left(4\;\mathrm{mg}/\mathrm{kg})\;x\;\mathrm{Body}\;\mathrm{Weight}\;(\mathrm{kg}\right)}{0.5\;\mathrm{mg}/\mathrm{mL}\;\mathrm{Stock}\;\mathrm{Solution}}$$

### 2.3 Non‐invasive peripheral FUS

**FIGURE 1:**
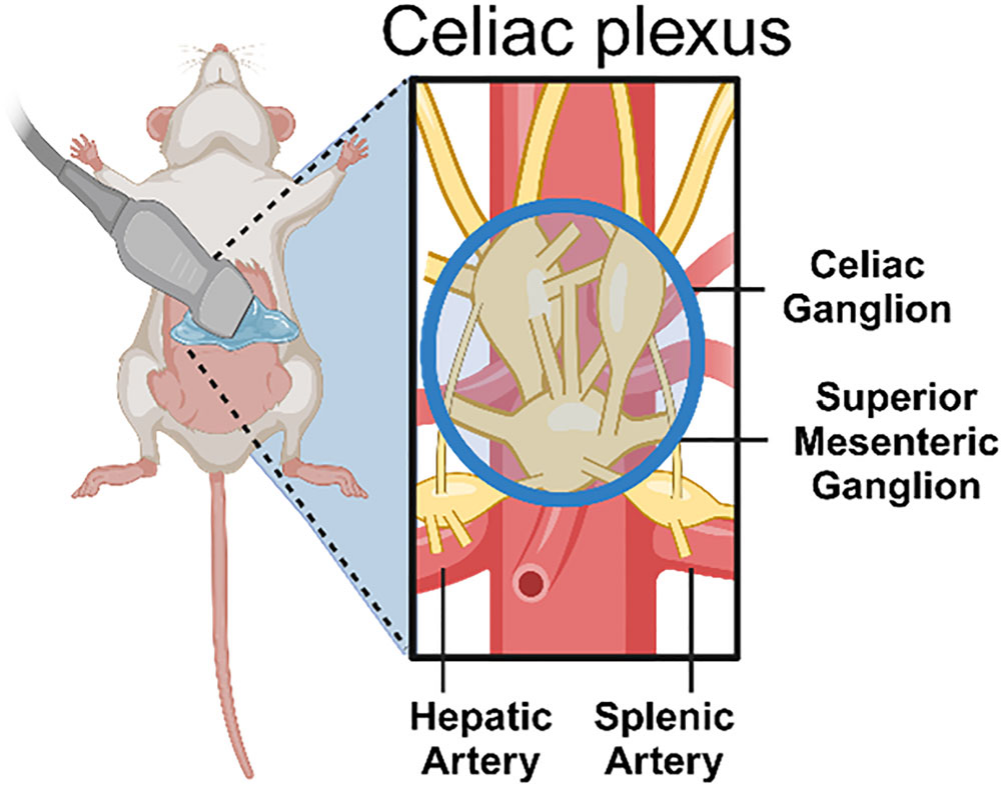
Focused ultrasound targeting of the celiac plexus in a rat model. Representative illustration of focused ultrasound (FUS) application targeting the celiac plexus on a supine rat. The left is a schematic illustration of a rodent, with the ultrasound transducer positioned on the abdominal surface to deliver FUS to the underlying celiac plexus. The right side provides an enlarged view of the celiac plexus, located around the abdominal aorta near the origin of the celiac trunk. The nerves of the celiac plexus (yellow) are shown in relation to the major blood vessels (red).

We implemented an FUS system comprising of a function generator (Agilent 33120A, Agilent Technologies, Santa Clara, CA, USA), a radio frequency ENI 350L power amplifier, and a custom‐made 2.5 MHz FUS transducer, as conducted in previous studies (Akhtar et al., 2021; Cotero et al., 2019). We targeted the celiac plexus, comprising the celiac and superior mesenteric ganglion, which contains both efferent and afferent neurons projecting to and innervating the intestines (McCorry, 2007), which makes it an ideal target for therapeutic FUS of the GI tract (Figure 1).

Rats were initially placed in an acrylic chamber for isoflurane‐mediated anaesthesia (1.5–2%). Once anaesthetized, animals were positioned in a nose cone to maintain continuous delivery of isoflurane and shaved in the epigastric abdominal region where ultrasound gel (Aquasonic, Fisher Scientific, Waltham, MA, USA) was applied to ensure optimal acoustic coupling. The FUS transducer probe was positioned in the left ventral area between the xiphoid process and the lower rib cage under isoflurane‐mediated anaesthesia previously identified by sonographic ultrasound (Akhtar et al., 2021). FUS was administered under isoflurane anaesthesia twice daily for 3 min, with 6‐h intervals between sessions, over five consecutive days. After 2 days, this was repeated in the same manner. Experimental control animals underwent the same anaesthesia and procedure, with the FUS machine remaining inactive.

### 2.4 Experimental timeline

**FIGURE 2:**
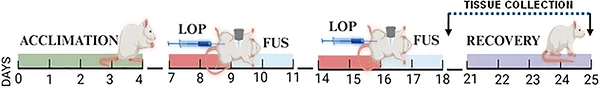
Experimental timeline to investigate the effects of peripheral focused ultrasound (FUS) on loperamide‐induced constipation. Single housed male Sprague–Dawley rats underwent acclimation, then two rounds peripheral ultrasound (FUS) or sham treatment concurrently with loperamide (LOP) or saline administration, followed by a recovery period.

The experimental design included four groups: saline, loperamide, saline–FUS and loperamide–FUS. Rats underwent an acclimation period from days 0 to 4, followed by peripheral FUS or sham treatments during two distinct phases: days 7–11 and days 14–18. Concurrently, loperamide or saline was administered on days 7, 8, 9, 14, 15 and 18. A subset of animals was retained for recovery following FUS treatment and monitored daily until day 25. Rats were sacrificed to collect tissue samples of the proximal colon (near the cecum) and distal colon (near the rectum) on either day 18 or day 25 for post‐mortem analysis (Figure 2).

### 2.5 Assessment of metabolic changes

To assess the effects of FUS on loperamide‐induced constipation, we examined metabolic changes including weight gain, food consumption and water intake daily (Ayari et al., 2025; Ju et al., 2020; Liu & Zhi, 2021; Wintola et al., 2010). Additionally, we monitored faecal pellet output, which is a common measure of constipation in loperamide‐treated rats (Kim et al., 2018; Wang et al., 2023; Wintola et al., 2010). Body weight changes were reported as a percentage relative to the initial body weight, using the formula:

$$\begin{array}{ccc} & & \mathrm{Body}\;\mathrm{Weight}\;\mathrm{Change}\;(\%)\\ & & \;=\left(\frac{\mathrm{Body}\;\mathrm{Weight}\;(g)\;-\;\mathrm{Initial}\;\mathrm{Body}\;\mathrm{Weight}\;(g)}{\mathrm{Initial}\;\mathrm{Body}\;\mathrm{Weight}\;(g)}\right)\times 100 \end{array}$$

Baseline food weight was taken on day 0 by removing the initial food from the home cage and placing it in a plastic container to be weighed. At the start of each subsequent day, the remaining food was weighed. To ensure ad libitum access to food, the chow was refilled weekly. Total consumption over 24 h was calculated as the difference between the initial weight and the remaining weight, expressed in grams, using the formula below:

$$\begin{array}{ccc} \mathrm{Food}\;\mathrm{Consumption}\;\left(g\right) & = & \mathrm{Initial}\;\mathrm{Food}\;\mathrm{Weight}\;\left(g\right)\\ & & -\;\mathrm{Subsequent}\;\mathrm{Food}\;\mathrm{Weight}\;(g) \end{array}$$

Baseline water weight in the bottle was recorded on day 0. At the start of each subsequent day, the weight of the water in the bottles was recorded. To ensure adequate access to water, the water bottles were replenished weekly. Total water consumption over a 24‐h period was calculated as the difference between the initial weight and the remaining weight, expressed in grams, using the formula below:

$$\begin{array}{ccc} \mathrm{Water}\;\mathrm{Consumption}\;\left(g\right) & = & \mathrm{Initial}\;\mathrm{Water}\;\mathrm{Weight}\;\left(g\right)\\ & & -\;\mathrm{Subsequent}\;\mathrm{Water}\;\mathrm{Weight}\;(g) \end{array}$$

### 2.6 Faecal pellet measurements

Throughout the experimental timeline, animals were individually housed in clean cages which were changed daily to facilitate the collection of faecal pellets. The cages were hand‐shifted daily to collect the faecal pellets and the total number of faecal pellets was recorded daily. Additionally, daily collected faecal pellets were dried and weighed for dry faecal weight to determine the total dry weight per pellet, providing insight into the effects of loperamide on increased water resorption and reduced transit time. To obtain the dry weight per pellet, faecal pellets were dried for at least 72 h to remove all moisture. Once dried, the total weight of the faecal pellets was recorded. The dry weight per pellet was then calculated by dividing the total dry weight of all collected pellets by the total number of pellets collected using the formula:

$$\mathrm{Dry}\;\mathrm{Weight}\;\mathrm{per}\;\mathrm{Pellet}\;(\mathrm{mg})=\left(\frac{\mathrm{Total}\;\mathrm{Dry}\;\mathrm{Weight}\;\mathrm{of}\;\mathrm{Pellets}\;(\mathrm{mg})}{\mathrm{Total}\;\mathrm{Number}\;\mathrm{of}\;\mathrm{Pellets}}\right)$$

### 2.7 Post‐mortem analyses

After experiments were completed, animals were anaesthetized with an intraperitoneal injection of urethane in saline (1.2–1.5 g/kg), sacrificed via a double thoracotomy, and dissected to remove the colon from the cecum to the anus. The colon was segmented into four sections: two proximal portions close to the caecum and two distal portions near the anus, each measuring ∼15–20 mm long. For post‐mortem analysis, one proximal and one distal colon segment were placed into sterile Eppendorf tubes, flash‐frozen in liquid nitrogen and stored at −80°C. The remaining proximal and distal segments from the same animal were fixed in paraformaldehyde for histological analysis.

### 2.8 Tissue lysate preparation

Proximal and distal colon tissue were separately homogenized using a lysis buffer (Tissue Extraction Reagent I, Thermo Fisher Scientific, Vienna, Austria) with an inhibitor cocktail and EDTA (Halt™ Protease Inhibitor Cocktail, Thermo Fisher Scientific, Waltham, MA, USA). The samples were centrifuged at 10,000 *g* for 30 min at 4°C and the supernatant was collected and stored at −80°C. Protein concentration was analysed using a bicinchoninic acid assay (BCA).

### 2.9 Chemiluminescence inflammation assay

**FIGURE 3:**
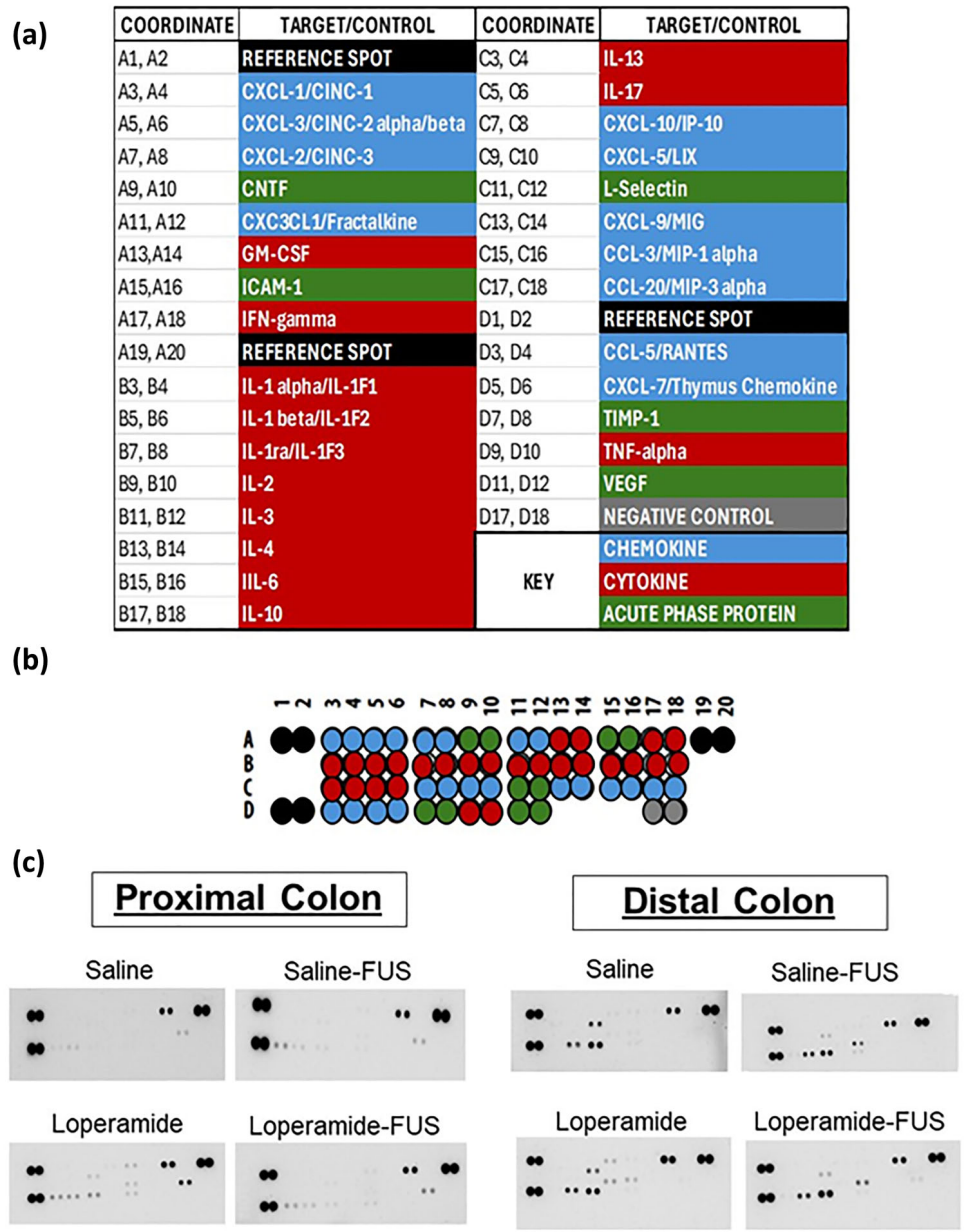
Chemiluminescence array blots with corresponding labels and representative images. (a) Table with coordinates and target/control labels corresponding to 29 different cytokines, chemokines and acute phase protein markers. (b) Representative array membrane layout. (c) Corresponding scanned images of the nitrocellulose membrane from the array kit of different animal groups. Darker dots, arising from the streptavidin–HRP and chemiluminescence detection reaction on the membranes represents protein expression levels from proximal or distal colon serum samples from animals in the saline, saline–FUS, loperamide and loperamide–FUS groups.

A Proteome Profiler Rat Cytokine Array Kit, Panel A (R&D systems, Minneapolis, MN, USA), a membrane‐based sandwich immunoassay, was used to assess inflammation in the distal and proximal colon of animals sacrificed on day 18 or 25. The array kit comprised control antibodies and 29 various inflammatory markers (cytokines (13), chemokines (11) and acute phase proteins (5)) in duplicates on a nitrocellulose membrane (Figure 3). A blocking buffer was added to a four‐well plate, and membranes were incubated for an hour. A solution containing 29 different biotinylated detection antibodies was incubated at room temperature for an hour. The membranes with tissue lysate were placed on a shaker (Microjive shaker, Boekel Scientific, Feasterville, PA, USA) and incubated overnight at −4°C. Membranes were then washed with the wash buffer to remove unbound material. Afterward, streptavidin–horseradish peroxidase (HRP) and chemiluminescent detection reagents were added to the membrane to be scanned using a digital block scanner (ChemiDoc Western Blot Digital imaging system, Bio‐Rad Laboratories, Hercules, CA, USA), resulting in the appearance of chemiluminescent spots. The position, size and intensity of these spots corresponded to the number of bound cytokines, chemokines and acute phase proteins. Membranes were analysed using HLImage++ (Western Vision Software, Salt Lake City, UT, USA).

### 2.10 Haematoxylin and eosin staining

Adjacent 1.5 cm segments of proximal and distal colon tissue were acquired for haematoxylin and eosin (H&E) staining to assess general histological structures, including the identification of inflammation, cell infiltration and tissue damage. During dissection, samples were immediately preserved in 4% paraformaldehyde. One week later, samples were transferred to 70% ethanol for processing and staining by the Albany Medical College Pathology Core. Tissue embedded in paraffin was transversely sectioned 5 µm thick and placed on charged microscope slides (Globe Scientific, Mahway, NJ, USA). Slides were dewaxed in xylene and washed three times, followed by a series of alcohol rinses at 100% ethanol (2×) and 95% ethanol (2×) to hydrate the tissue. The slides were placed in haematoxylin 7211, differentiated using a blue reagent bath with Eosin‐Y and then underwent a 95% alcohol wash. Finally, slides were dehydrated in 100% ethanol wash and cleared with a xylene substitute wash before being cover‐slipped with Cytoseal 60 (Fisher Scientific, Waltham, MA, USA).

The H&E‐stained sections were used to assess epithelial damage and infiltration of the proximal and distal colon sections using a semi‐quantitative scoring rubric by two pathologists (Chen et al., 2019; Takagawa et al., 2018; Wirtz et al., 2017). Following the scoring rubric, a total histopathological damage score for each section of the colon was obtained by summing the individual scores (0–4) for epithelial damage (0: normal morphology, intact goblet cells and crypt structures; 1: loss of goblet cells; 2: goblet cells lost across large areas; 3: loss of crypts, severe disruption in the epithelial architecture; 4: extensive loss of crypts in large areas) and infiltration (0: no infiltration and normal tissue morphology; 1: infiltration localized near crypt base; 2: inflammatory cells in the muscularis mucosae; 3: extensive infiltrations in the muscularis mucosae, the presence of oedema and thickening of the mucosa; 4: inflammatory cells penetrating the submucosa) to provide a comprehensive measure of the overall histopathological changes (0–8) that reflects the severity of tissue damage and inflammation in the colon (Akhtar et al., 2021).

### 2.11 Statistical analysis

Data were analysed using a two‐way analysis of variance (ANOVA) in GraphPad Prism (V. 10.0.2; GraphPad Software, Boston, MA, USA). All data were presented as the means ± standard deviation (SD) with statistical significance set at *P <* 0.05 for comparisons between saline, saline–FUS, loperamide and loperamide–FUS groups. Sample sizes for multiple groups were reported using a forward slash to delineate between the described groups. If data points were missing, a repeated‐measures mixed‐effects model (RMEM) was used, followed by Tukey's multiple comparisons test. Differences between two groups were assessed using Student's *t*‐test.

Cytokine, chemokine and acute phase reactant expression was quantified by measuring the mean pixel density of the chemiluminescent stain. The recorded mean pixel densities of duplicate membrane blots were averaged and normalized against the mean pixel density of negative control values. A two‐way ANOVA with Fisher's least significant difference (LSD) *post hoc* analysis was used to assess differences in inflammatory marker expression in the distal and proximal colon after FUS treatment and following a 1‐week recovery period.

## 3 RESULTS

**FIGURE 4:**
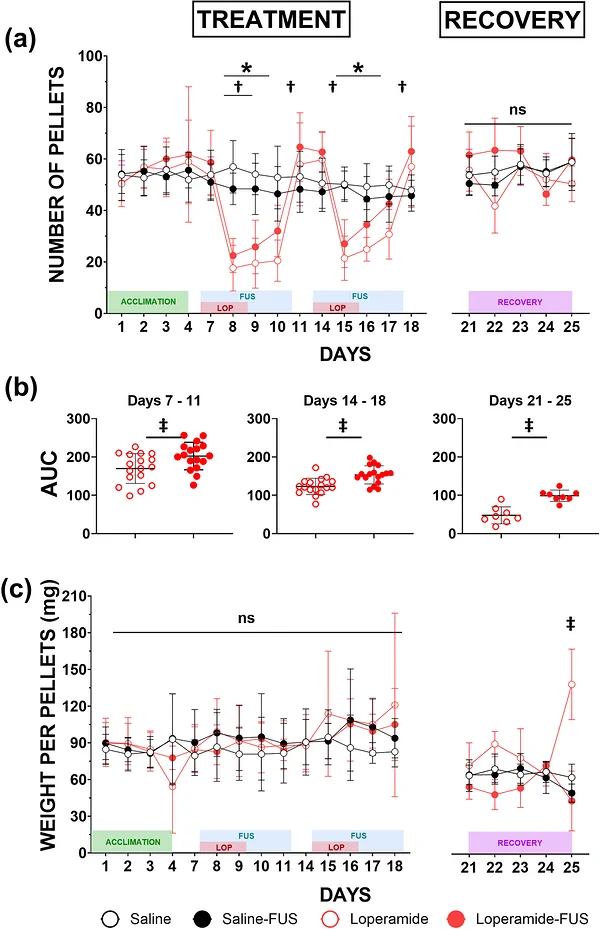
Effects of FUS on faecal pellet output in loperamide‐induced constipation. Faecal pellet outputs were collected daily from individual home cages. (a) Number of faecal pellet output daily. (b) The area under the curve (AUC) for number of weekly faecal pellets. (c) 72 h dry weight per pellet in milligrams (mg). Data presented as means ± SD. Statistical analyses were conducted using a repeated‐measures mixed‐effects model (RMEM) followed by Tukey's multiple comparisons test (a, c) or paired Student's *t*‐test (b): *loperamide vs. saline; †loperamide–FUS vs. saline–FUS; ‡loperamide–FUS vs. loperamide: *P <* 0.05.

The number of faecal pellets decreased between loperamide (*n* = 17) and saline (*n* = 14) groups on days 8, 9, 10, 15, 16 (*P <* 0.001) and 17 (*P = *0.031) (Figure 4a), as well as between loperamide–FUS (*n* = 17) and saline–FUS (*n* = 17) rats on days 8 (*P <* 0.001), 9 (*P <* 0.001), 11 (*P = *0.010), 15 (*P <* 0.001) and 18 (*P = *0.004) (Figure 4a). Notably, FUS increased faecal pellet output with higher area under the curve (AUC) values in animals in the loperamide–FUS cohort (*n* = 17) compared to loperamide (*n* = 17) during both FUS treatment (days 7–11 and 14–18) and recovery (days 21–25) (Figure 4b). Loperamide‐treated animals also had heavier faecal pellets than both saline and loperamide–FUS‐treated (*n* = 8) animals during the recovery period on day 25 (*P <* 0.001) (Figure 4c).

**FIGURE 5:**
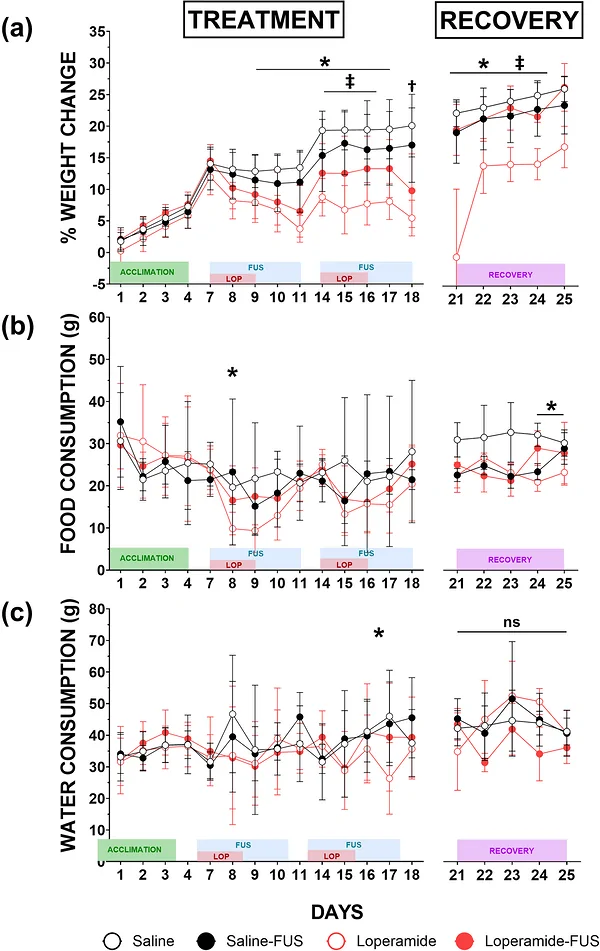
Effects of FUS on metabolic changes in loperamide‐induced constipation. Animal body, food and water weights from individual home cages were recorded daily. The effects of FUS on these parameters were assessed by calculating (a) percent (%) weight change, (b) daily food, and (c) water consumption of rats in each group. Data presented as means ± SD. Statistical analyses were conducted using a RMEM followed by Tukey's multiple comparisons test: *loperamide vs. saline; †loperamide–FUS vs. saline–FUS; ‡loperamide–FUS vs. loperamide: *P <* 0.05.

Animals exhibited less weight change in the loperamide‐treatment group (*n* = 17) than the saline‐treatment group (*n* = 14) on days 10–18 (*P <* 0.001) (Figure 5a). A decrease in percent weight change was also observed during the recovery period between loperamide (*n* = 8) and saline groups (*n* = 7) on days 21 (*P <* 0.001), 22 (*P = *0.001), 23 (*P = *0.002), 24 (*P <* 0.001) and 25 (*P = *0.009) (Figure 5a). The application of FUS resulted in a decrease in weight change on day 18 (*P <* 0.001) in the loperamide–FUS (*n* = 17) and saline–FUS groups (*n* = 14) (Figure 5a). Compared to loperamide without FUS treatment, loperamide–FUS‐treated rats had increased percent weight change on days 15 (*P = *0.004), 16 (*P = *0.007) and 17 (*P = *0.024) (Figure 5a). The increased percent weight change from FUS was observed during the recovery period for loperamide–FUS‐treated rats (*n* = 8) on days 21 (*P <* 0.001), 22, (*P = *0.009), 23, (*P = *0.008), 24 (*P = *0.044) and 25 (*P = *0.009) when compared to loperamide without treatment groups (Figure 5a).

For food consumption, there was no difference between loperamide‐treated and saline‐treated rats for days 1–18 with an exception on day 8 (*P <* 0.05). However, loperamide treatment decreased food consumption during the recovery periods between days 24 (*P <* 0.001) and 25 (*P = *0.046) compared to saline‐treated animals (Figure 5c).

**FIGURE A1:**
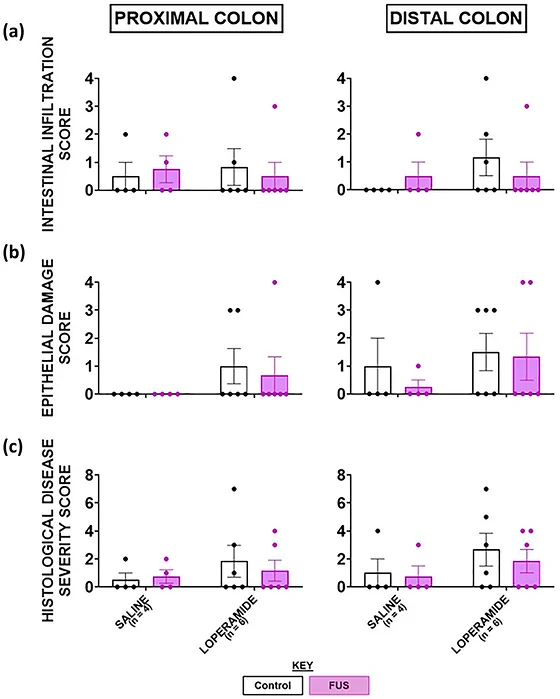
FUS did not impact colon morphology in loperamide‐induced constipation at day 18. Proximal and distal colon samples were collected on day 18 from each animal, and haematoxylin and eosin staining was used for sections. (a, b) Intestinal infiltration (a) and epithelial damage (b) were scored on a rating scale (1–4). (c) The sum of both scores represents the total histological disease severity score (0–8) of the proximal or distal colon. Each dot represents a single animal. Data are presented as means ± SEM. No significant differences were noted using a two‐way ANOVA followed by Tukey's multiple comparisons test.

**FIGURE A2:**
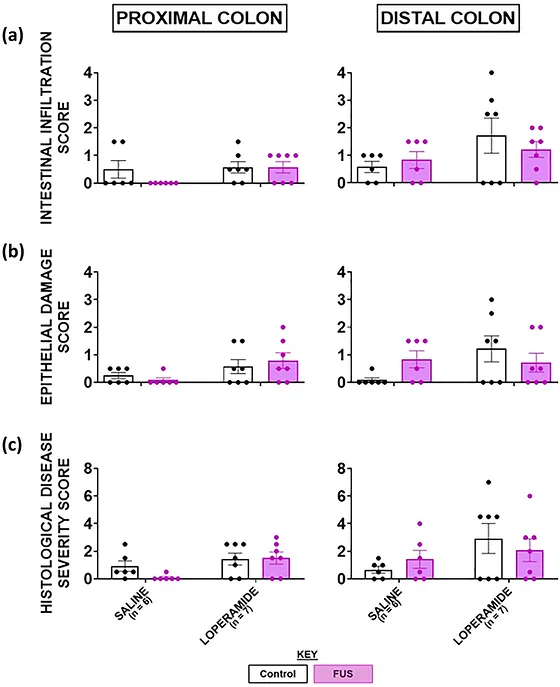
FUS did not impact colon morphology in loperamide‐induced constipation at day 25. Proximal and distal colon samples were collected on day 25 from each animal, and haematoxylin and eosin staining was used for sections. (a, b) Intestinal infiltration (a) and epithelial damage (b) were scored on a rating scale (1–4). (c) The sum of both scores represents the total histological disease severity score (0–8) of the proximal or distal colon. Each dot represents a single animal. Data are presented in means ± SEM. No statistical differences were noted using a two‐way ANOVA followed by Tukey's multiple comparisons test.

Proximal and distal colon tissue did not exhibit any differences in intestinal infiltration, epithelial damage and histological disease severity scores on day 18 (Appendix Figure A1) and day 25 (Figure A2) between any groups.

**FIGURE 6:**
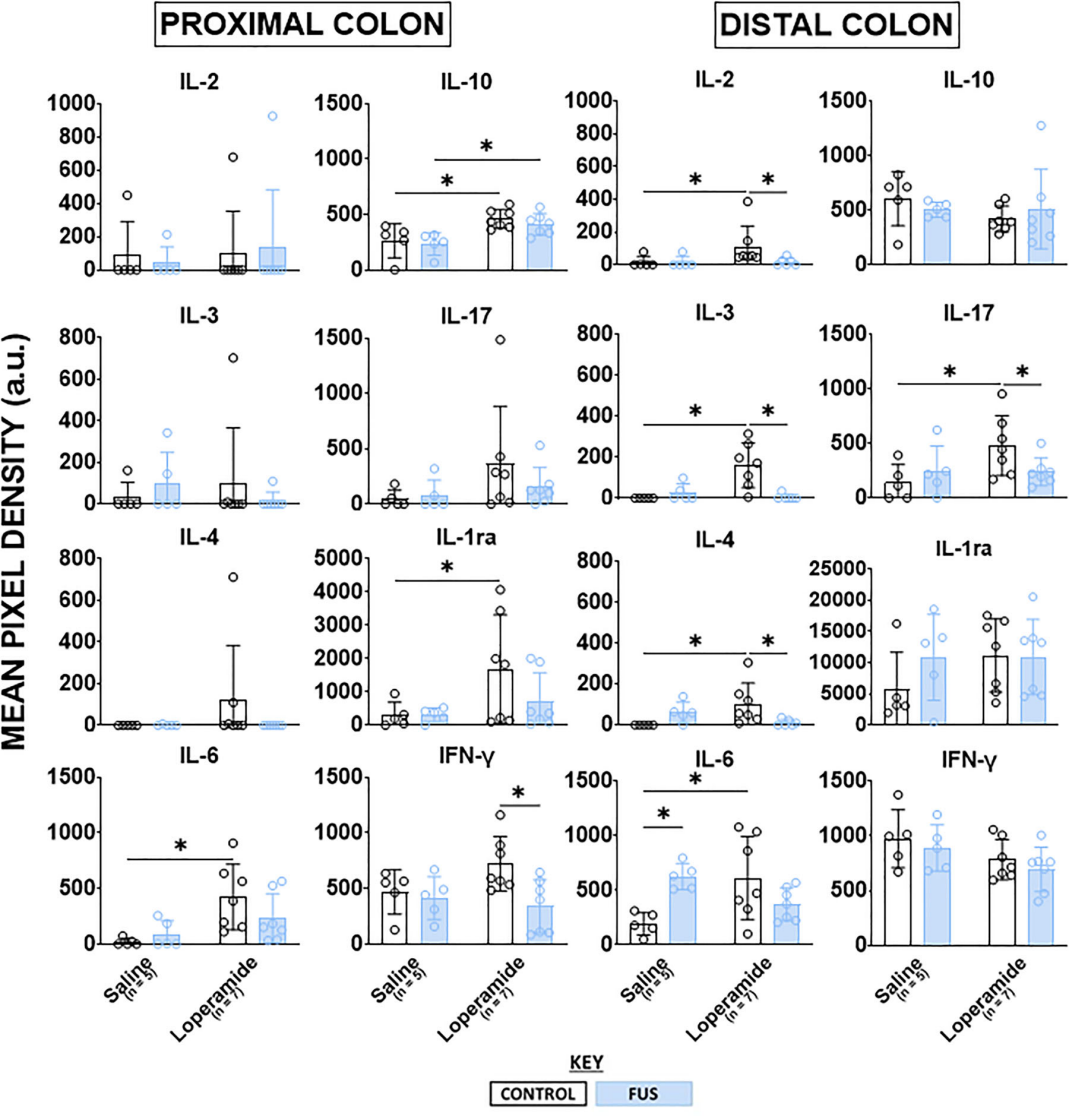
Impact of FUS on colonic cytokine expression of loperamide‐induced constipation at day 18. Proximal and distal colon samples were collected on day 18 from each animal and assessed for cytokine mean pixel density expression following a chemiluminescence array. Each dot represents a single animal. Data are presented as means ± SD. Statistical analyses were conducted using a two‐way ANOVA with Fisher's least significant difference (LSD) test: *P < 0.05.

**FIGURE A3:**
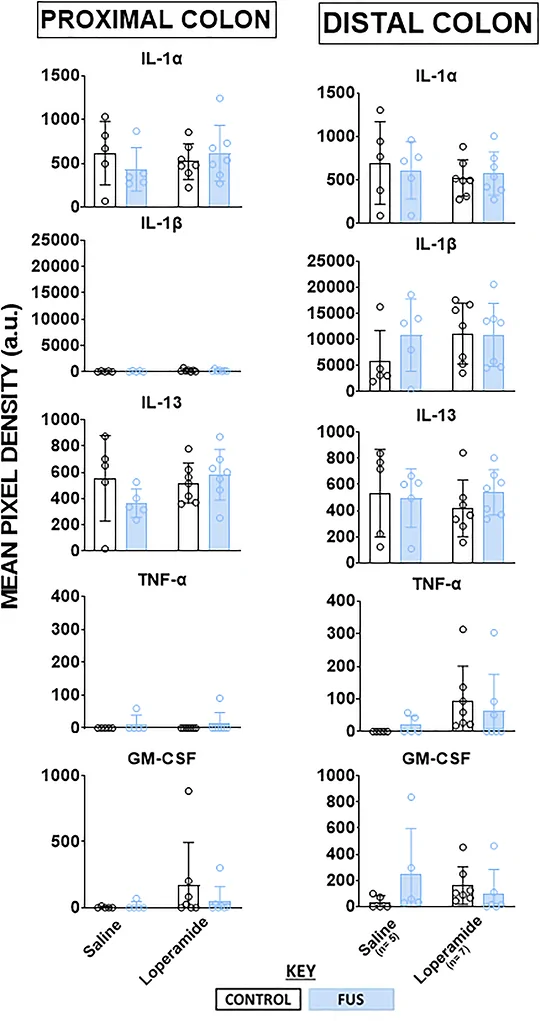
Colonic cytokine expression showing no significant differences in loperamide‐induced constipation at day 18. Proximal and distal colon samples were collected on day 18 from each animal and assessed for cytokine mean pixel density expression following a chemiluminescence array. Each dot represents a single animal. Data are presented as means ± SD. Statistical analyses were conducted using a two‐way ANOVA with Fisher's least significant difference (LSD) test. Statistically significant changes were not observed between any groups.

Using tissue lysate from both the proximal and distal colon from rats in all four experimental groups (saline, loperamide, saline–FUS, loperamide–FUS), we employed a chemiluminescence array to detect the expression of 13 different cytokines on day 18. Loperamide‐treated rats (*n* = 7) had increased expression of proximal colon cytokines IL‐10 (*P = *0.006) and IL‐1ra (*P = *0.032) compared to saline‐treated rats (*n* = 5) (Figure 6). In the distal colon, cytokines IL‐2 (*P = *0.045), IL‐3 (*P <* 0.001), IL‐4 (*P = *0.011) and IL‐17 (*P = *0.012) also showed elevated expression in loperamide‐treated animals (Figure 6). An increase in IL‐6 expression was observed in both the proximal (*P = *0.004) and distal (*P = *0.006) colons of rats treated with loperamide compared to those treated with saline (Figure 6). While testing the therapeutic effects of FUS on loperamide‐induced constipation, we only found one proximal colon cytokine with an increase in loperamide–FUS‐treated rats (*n* = 7) compared to saline–FUS‐treated rats (*n* = 5), which was IL‐10 (*P = *0.011) (Figure 6). FUS treatment reduced the distal colon cytokines IL‐2 (*P = *0.038), IL‐3 (*P = *0.038), IL‐4 (*P = *0.017) and IL‐17 (*P = *0.045) in loperamide–FUS‐treated animals compared to those with loperamide alone (Figure 6). FUS also led to a significant reduction in the expression of proximal interferon (IFN)‐γ (*P = *0.005) in loperamide–FUS‐treated animals (Figure 6). In contrast, FUS increased distal colon IL‐6 (*P = *0.008) cytokine expression in saline–FUS‐treated animals compared to animals given saline alone (Figure 6). No difference was found in IL‐13, IL‐1α, IL‐1β, tumour necrosis factor α (TNF‐α) and granulocyte–macrophage colony‐stimulating factor (GM‐CSF) cytokine expression at the proximal or distal colon for loperamide‐treated and FUS‐treated cohorts at day 18 (*P >* 0.050; Figure A3).

**FIGURE 7:**
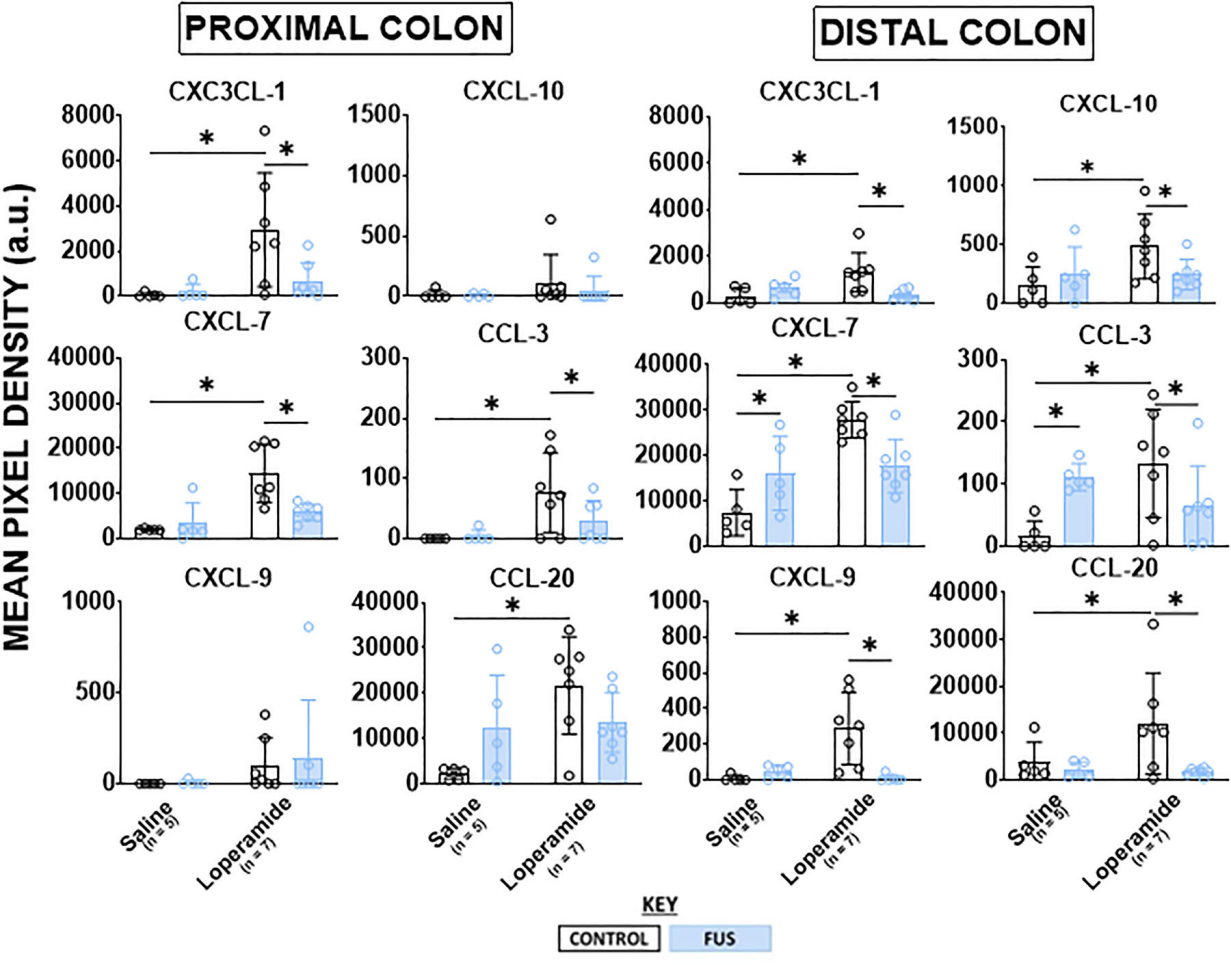
Impact of FUS on colonic chemokine expression in loperamide‐induced constipation at day 18. Proximal and distal colon samples were collected on day 18 from each animal and assessed for chemokine mean pixel density expression following a chemiluminescence array. Each dot represents a single animal. Data are presented as means ± SD. Statistical analyses were conducted using a Two‐Way ANOVA with Fisher's LSD test: *P < 0.05.

**FIGURE A4:**
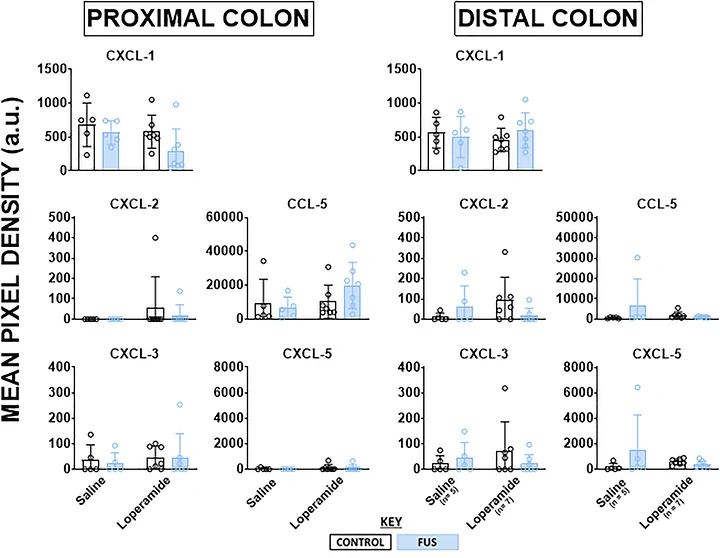
Colonic chemokine expression showing no significant differences in loperamide‐induced constipation at day 18. Proximal and distal colon samples were collected on day 18 from each animal and assessed for chemokine mean pixel density expression following a chemiluminescence array. Each dot represents a single animal. Data are represented in means ± SD. Statistical analyses were conducted using a two‐way ANOVA with Fisher's LSD test. Statistically significant changes were not observed between any groups.

On day 18, loperamide (*n* = 7) treatment resulted in an increase in chemokine expression in the proximal colon compared to saline treatment (*n* = 5), with elevated levels of CXC3CL‐1 (*P = *0.003), CXCL‐7 (*P = *0.003), CCL‐3 (*P = *0.005) and CCL‐20 (*P = *0.001) (Figure 7). In the distal colon, upregulation was also observed for CXC3CL‐1 (*P = *0.003), CXCL‐7 (*P <* 0.001), CCL‐3 (*P = *0.004), CCL‐20 (*P = *0.035), along with CXCL‐9 (*P <* 0.001) and CXCL‐10 (*P = *0.012) in loperamide‐treated compared to saline‐treated rats (Figure 7). Following FUS treatment, loperamide‐treated (*n* = 7) animals exhibited a decrease in chemokine expression in the proximal colon (CXC3CL‐1 (*P = *0.009), CXCL‐7 (*P = *0.001), CCL‐3 (*P = *0.042)) and distal colon (CXC3CL‐1 (*P = *0.002), CXCL‐7 (*P = *0.004), CCL‐3 (*P = *0.049), CCL‐20 (*P = *0.006), CXCL‐9 (*P <* 0.001), CXCL‐10 (*P = *0.045)) compared to the loperamide‐treated cohort (Figure 7). Saline–FUS‐treated (*n* = 5) animals had an increase in expression of distal colon chemokines CXCL‐7 (*P = *0.029) and CCL‐3 (*P = *0.022) compared to saline‐only rats (Figure 7). CXCL‐1, CXCL‐2, CXCL‐3, CXCL‐5 and CCL‐5 chemokine expression were no different at the proximal and distal colon for loperamide‐ and FUS‐treated animals at day 18 (*P >* 0.050; Figure A4).

**FIGURE 8:**
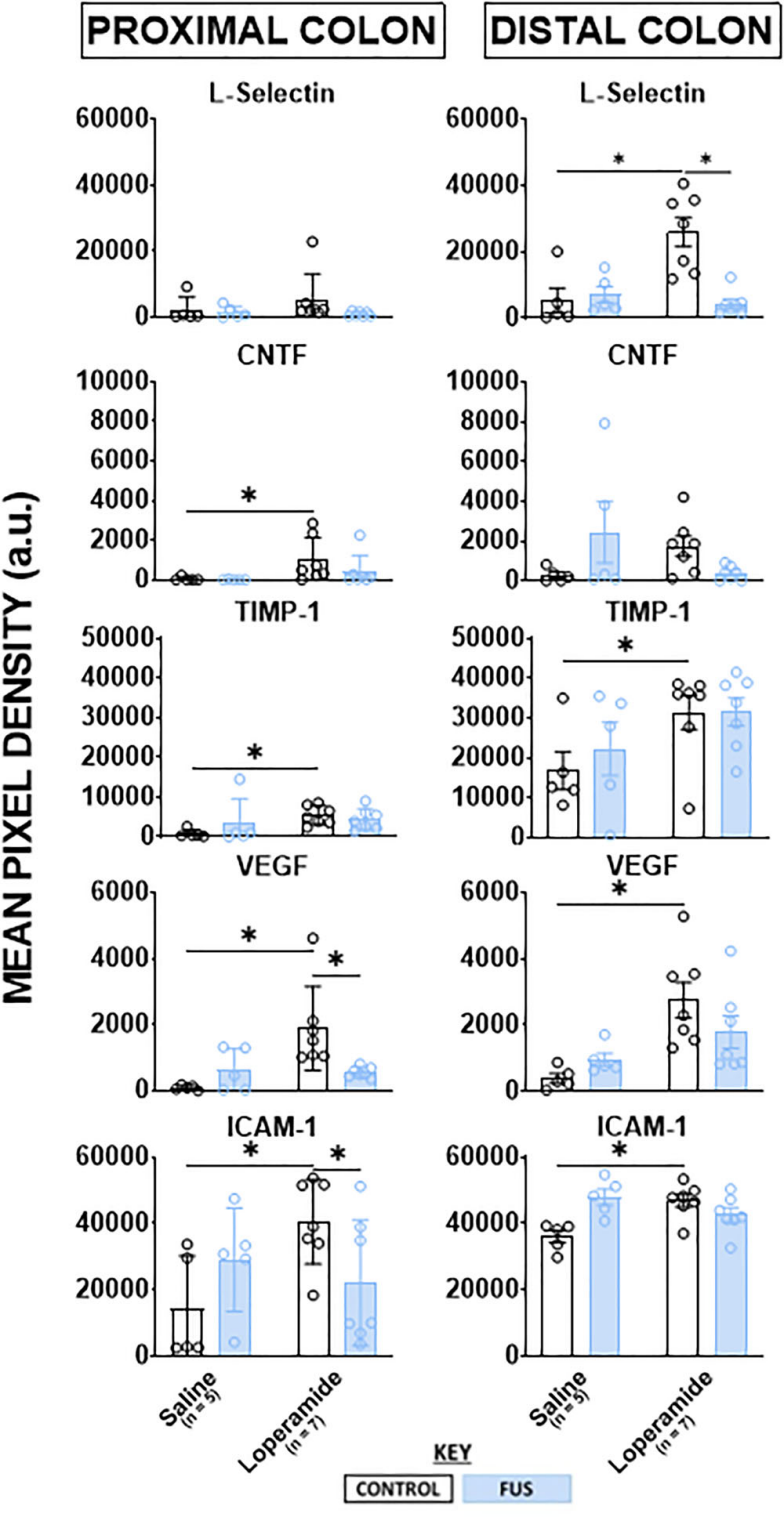
Impact of FUS on colonic acute phase protein expression in loperamide‐induced constipation at day 18. Proximal and distal colon samples were collected on day 18 from each animal and assessed for acute phase protein mean pixel density expression following a chemiluminescence array. Each dot represents a single animal. Data are presented as means ± SD. Statistical analyses were conducted using a two‐way ANOVA with Fisher's LSD test: *P < 0.05.

Loperamide‐treated rats (*n* = 7) had an increase in the proximal colon expression of acute phase proteins, specifically ciliary neurotrophic factor (CNTF) (*P = *0.047), tissue inhibitor of metalloproteinases (TIMP)‐1 (*P = *0.031), vascular endothelial growth factor (VEGF) (*P <* 0.001) and intercellular adhesion molecule (ICAM)‐1 (*P = *0.011) when compared to saline‐treated rats (*n* = 5) (Figure 8). The distal colon also exhibited increased expression of l‐selectin (*P <* 0.001), TIMP‐1 (*P = *0.042), VEGF (*P = *0.001) and ICAM‐1 (*P = *0.002) in the colon of loperamide‐treated animals (Figure 8). FUS treatment decreased proximal colon ICAM‐1 (*P = *0.045) and VEGF (*P = *0.004) in loperamide‐treated animals (*n* = 7) compared to those administered loperamide without FUS treatment. Additionally, l‐selectin (*P <* 0.001) showed a decrease in the distal colon of loperamide–FUS‐treated animals compared to loperamide‐treated rats (Figure 8). Of note, FUS treatment increased distal colon ICAM‐1 (*P = *0.002) in saline–FUS‐treated animals (*n* = 5) compared to animals given saline without FUS treatment (Figure 8). A summary of all day 18 inflammatory marker data for rats treated with saline and loperamide with and without FUS treatment is provided in Table 1.

**TABLE 1:** Summary of changes in inflammatory markers for rats treated with saline and loperamide with and without FUS treatment on day 18.

	Proximal colon				Distal colon			
	Effect of loperamide		Effect of FUS		Effect of loperamide		Effect of FUS	
Inflammatory markers	−FUS	+FUS	Within saline groups	Within loperamide groups	−FUS	+FUS	Within saline groups	Within loperamide groups
**IL‐2**	—	—	—	—	↑	—	—	↓
**IL‐10**	↑	↑	—	—	—	—	—	—
**IL‐3**	—	—	—	—	↑	—	—	↓
**IL‐17**	—	—	—	—	↑	—	—	↓
**IL‐4**	—	—	—	—	↑	—	—	↓
**IL‐1ra**	↑	—	—	—	—	—	—	—
**IL‐6**	↑	—	—	—	↑	—	↑	—
**IFN‐γ**	—	—	—	↓	—	—	—	—
**IL‐1α**	—	—	—	—	—	—	—	—
**IL‐1β**	—	—	—	—	—	—	—	—
**IL‐13**	—	—	—	—	—	—	—	—
**TNF‐α**	—	—	—	—	—	—	—	—
**GM‐CSF**	—	—	—	—	—	—	—	—
**CXC3CL‐1**	↑	—	—	↓	↑	—	—	↓
**CXCL‐10**	—	—	—	—	↑	—	—	↓
**CXCL‐7**	↑	—	—	↓	↑	—	↑	↓
**CCL‐3**	↑	—	—	↓	↑	—	↑	↓
**CXCL‐3**	—	—	—	—	—	—	—	—
**CXCL‐5**	—	—	—	—	—	—	—	—
**CCL‐5**	—	—	—	—	—	—	—	—
**CXCL‐1**	—	—	—	—	—	—	—	—
**CXCL‐2**	—	—	—	—	—	—	—	—
**CXCL‐9**	—	—	—	—	↑	—	—	↓
**CCL‐20**	↑	—	—	—	↑	—	—	↓
**L‐Selectin**	—	—	—	—	↑	—	—	↓
**CNTF**	↑	—	—	—	—	—	—	—
**TIMP‐1**	↑	—	—	—	↑	—	—	—
**VEGF**	↑	—	—	↓	↑	—	—	—
**ICAM‐1**	↑	—	—	↓	↑	—	—	—

An arrow indicates significance, and the direction denotes an increase or decrease based on saline compared to loperamide and without FUS compared to with FUS treatment.

**FIGURE 9:**
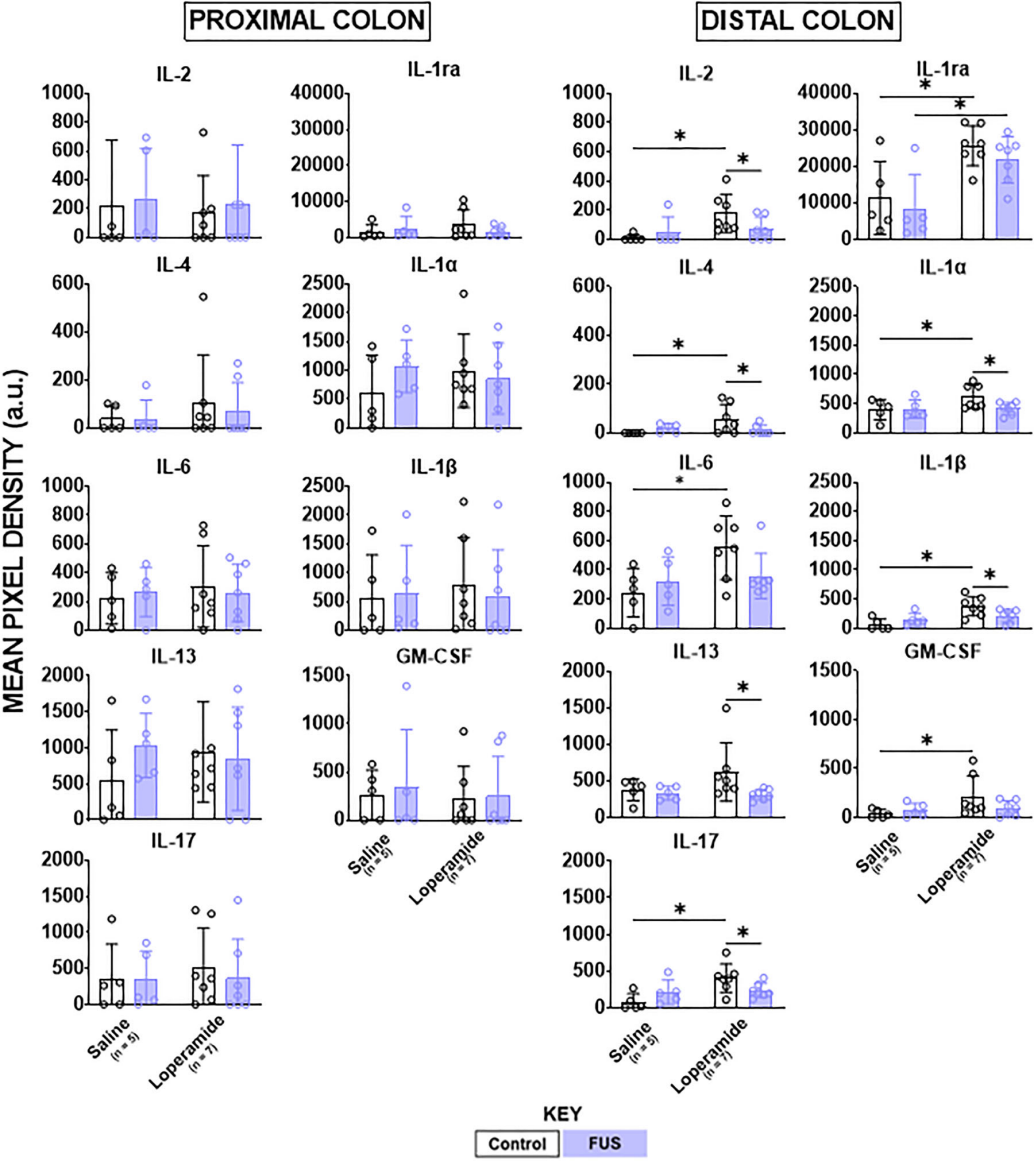
Impact of FUS on colonic cytokine expression in loperamide‐induced constipation at day 25. Proximal and distal colon samples were collected on day 25 from each animal and assessed for cytokine mean pixel density expression following a chemiluminescence array. Each dot represents a single animal. Data are presented as means ± SD. Statistical analyses were conducted using a two‐way ANOVA with Fisher's LSD test: *P < 0.05.

**FIGURE A5:**
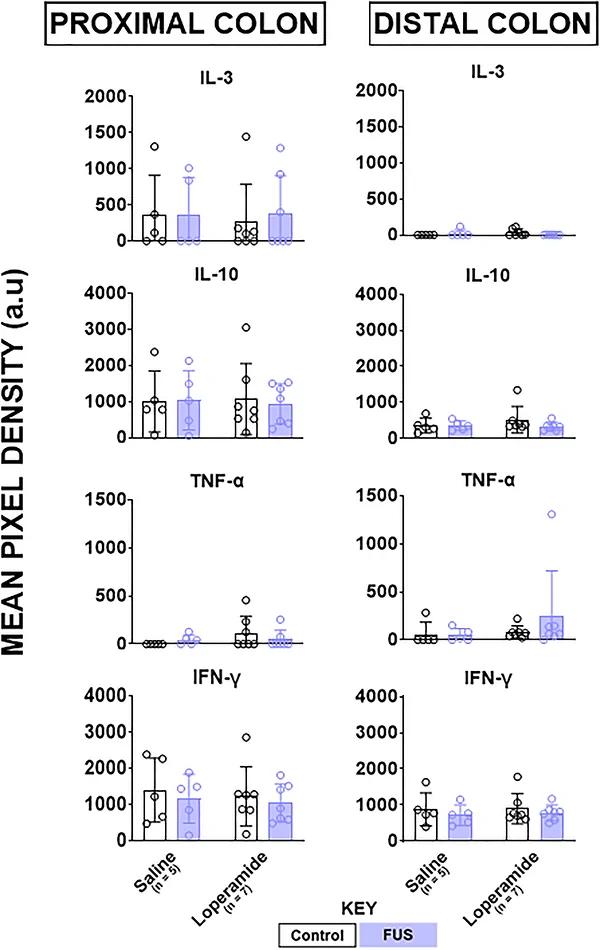
Colonic cytokine expression showing no significant differences in loperamide‐induced constipation at day 25. Proximal and distal colon samples were collected on day 25 from each animal and assessed for cytokine mean pixel density expression following a chemiluminescence array. Each dot represents a single animal. Data are presented as means ± SD. Statistical analyses were conducted using a two‐way ANOVA with Fisher's LSD test. Statistically significant changes were not observed between any groups.

To evaluate the effects of loperamide and FUS treatment, animals recovered for 1 week and were sacrificed on day 25. Rats treated with loperamide (*n* = 7) had an increase in expression of eight distal colon cytokines: IL‐2 (*P = *0.008), IL‐4 (*P = *0.015), IL‐6 (*P = *0.009), IL‐17 (*P = *0.001), IL‐1ra (*P = *0.005), IL‐1α (*P = *0.035), IL‐1β (*P <* 0.001) and GM‐CSF (*P = *0.030), when compared to saline‐treated animals (*n* = 5) (Figure 9). Loperamide‐treated rats receiving FUS treatment (*n* = 7) had a decrease in expression of six different distal colon cytokines, IL‐2 (*P = *0.049), IL‐4 (*P = *0.031), IL‐13 (*P = *0.024), IL‐17 (*P = *0.047), IL‐1α (*P = *0.036) and IL‐1β (*P = *0.025), compared to loperamide animals without FUS treatment (Figure 9). Additionally, loperamide–FUS‐treated animals exhibited an increase in distal colon IL‐1ra expression (*P = *0.007) compared to the saline–FUS group (*n* = 5), with no other cytokines altered between the two groups (Figure 9). No differences in cytokine expression were found in the proximal colon (Figure 9). IL‐3, IL‐10, TNF‐α and IFN‐γ revealed no difference in cytokine expression at the proximal or distal colon for loperamide‐treated and FUS‐treated cohorts at day 25 (*P >* 0.050; Figure A5).

**FIGURE 10:**
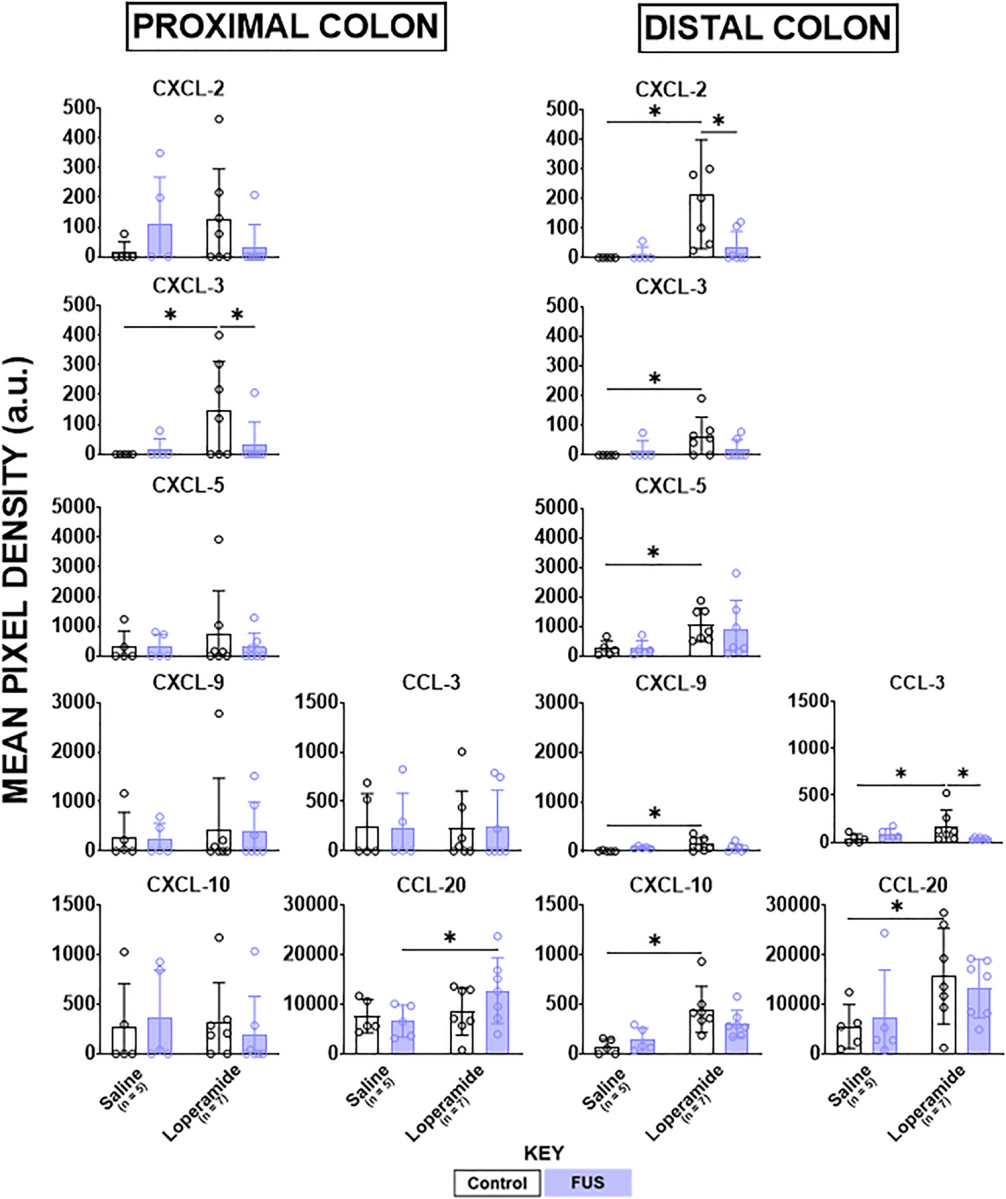
Impact of FUS on colonic chemokine expression in loperamide‐induced constipation at day 25. Proximal and distal colon samples were collected on day 25 from each animal and assessed for chemokine mean pixel density expression following a chemiluminescence array. Each dot represents a single animal. Data are presented as means ± SD. Statistical analyses were conducted using a two‐way ANOVA with Fisher's LSD test: *P < 0.05.

**FIGURE A6:**
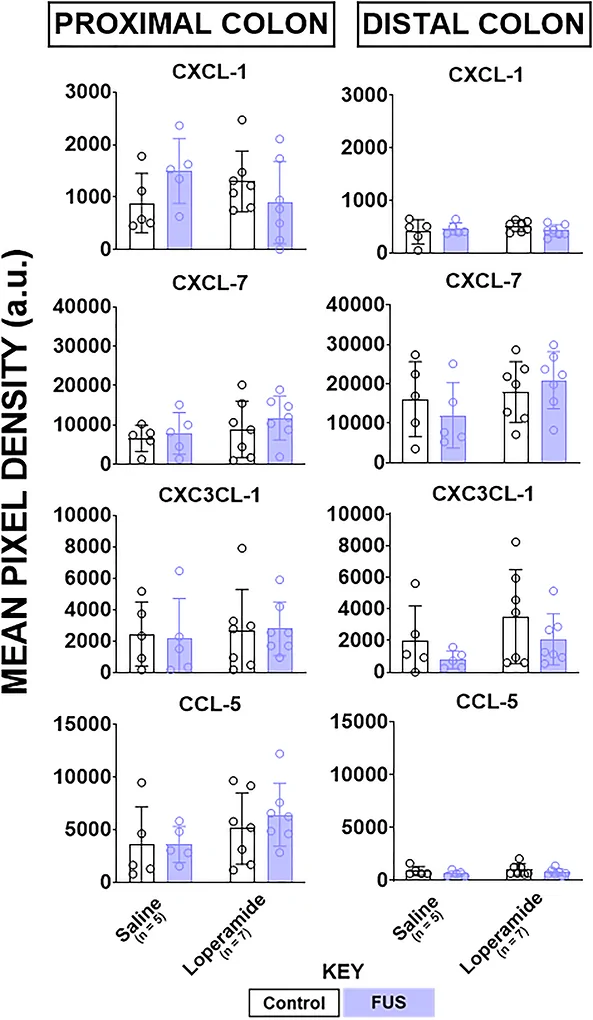
Colonic chemokine expression showing no significant differences in loperamide‐induced constipation. Proximal and distal colon samples were collected on day 25 from each animal and assessed for chemokine mean pixel density expression following a chemiluminescence array. Each dot represents a single animal. Data are presented as means ± SD. Statistical analyses were conducted using a two‐way ANOVA with Fisher's LSD test. Statistically significant changes were not observed between any groups.

After one week of recovery, on day 25, loperamide‐treated animals (*n* = 7) had increased expression of the proximal colon chemokine CXCL‐3 (*P = *0.020) compared to saline‐treated rats (*n* = 5). In the distal colon of rats treated with loperamide, seven distinct chemokines increased expression, including CXCL‐2 (*P = *0.002), CXCL‐3 (*P = *0.021), CXCL‐5 (*P = *0.047), CXCL‐9 (*P = *0.013), CXCL‐10 (*P <* 0.001), CCL‐3 (*P = *0.042) and CCL‐20 (*P = *0.038) (Figure 10). FUS treatment resulted in a decrease in expression of one proximal colon and two distal colon chemokines. Loperamide–FUS‐treated animals (*n* = 7) had a decrease of CXCL‐3 (*P = *0.038) in the proximal colon, and CXCL‐2 (*P = *0.004) and CCL‐3 (*P = *0.024) in the distal colon, compared to loperamide‐treated rats without FUS. An increase in chemokine CCL‐20 expression in the proximal colon (*P = *0.046) was seen between loperamide–FUS compared to the saline–FUS‐treated groups (*n* = 5) (Figure 10). Expression of chemokines CXCL‐1, CXCL‐7, CXC3CL‐1 and CCL‐5 was no different in the proximal or distal colon for loperamide‐treated and FUS‐treated cohorts at day 25 (*P >* 0.050; Figure A6).

**FIGURE 11:**
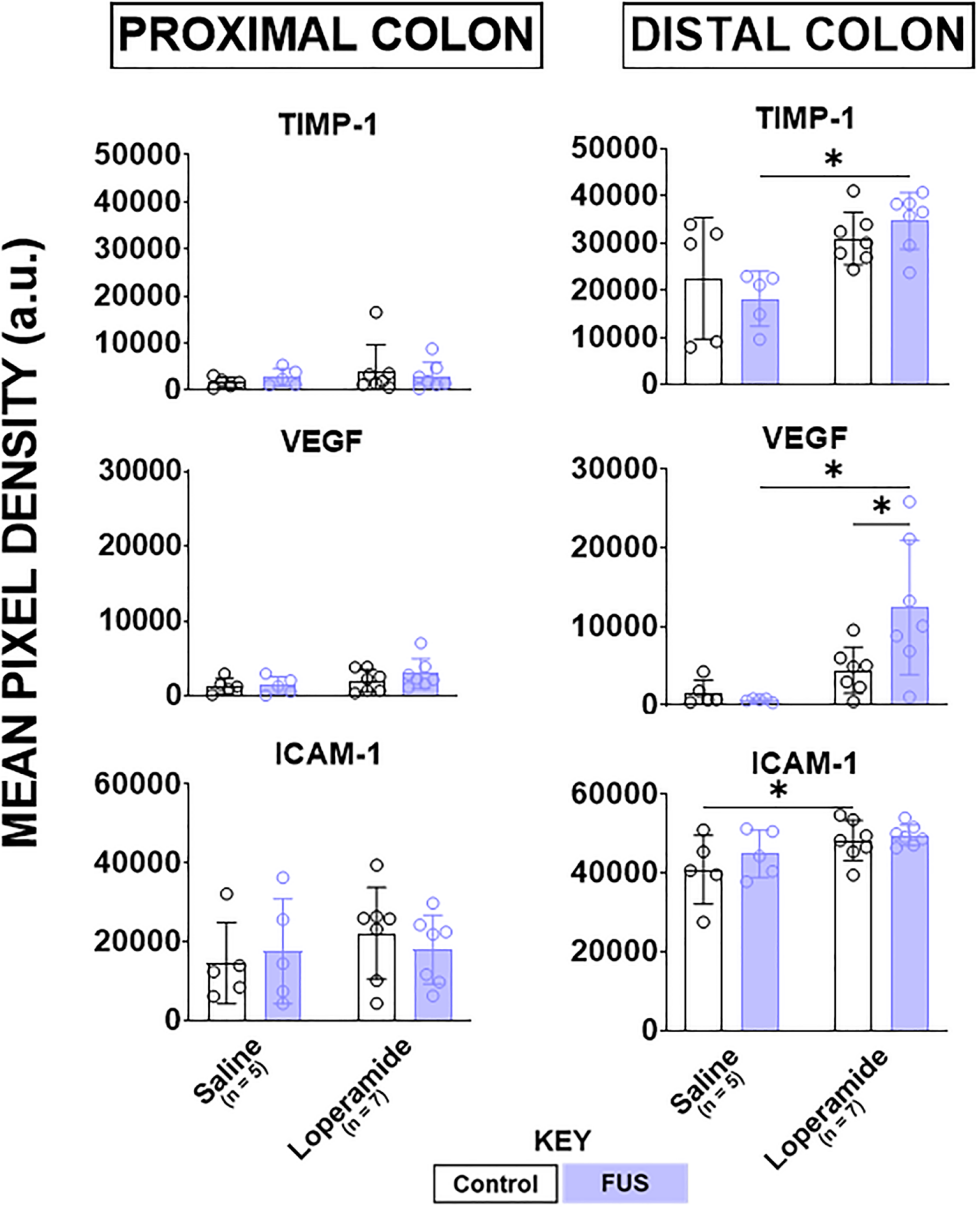
Impact of FUS on colonic acute phase proteins expression in loperamide‐treated rats up to 1‐week post‐treatment at day 25. Proximal and distal colon samples were collected on day 25 from each animal and assessed for acute phase protein mean pixel density expression following a chemiluminescence array. Each dot represents a single animal. Data are presented as means ± SD. Statistical analyses were conducted using a two‐way ANOVA with Fisher's LSD test: *P < 0.05.

**FIGURE A7:**
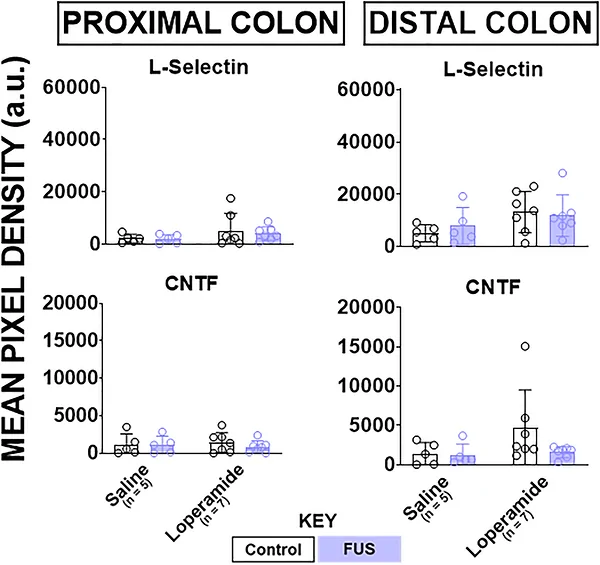
Colonic acute phase protein expression showing no significant differences in loperamide‐induced constipation on day 25. Proximal and distal colon samples were collected on day 25 from each animal and assessed for acute phase protein mean pixel density expression following a chemiluminescence array. Each dot represents a single animal. Data are presented as means ± SD. Statistical analyses were conducted using a two‐way ANOVA with Fisher's LSD test. Statistically significant changes were not observed between any groups.

Loperamide‐treated animals (*n* = 7) exhibited an increase in distal colon ICAM‐1 (*P = *0.039) expression compared to saline‐treated rats (*n* = 5) (Figure 11). An increase in expression of distal colon VEGF (*P = *0.007) was observed between loperamide‐ and loperamide–FUS‐treated (*n* = 7) groups, with FUS resulting in more VEGF expression. Increased expression of the distal colon chemokines VEGF (*P <* 0.001) and TIMP‐1 (*P = *0.002) in loperamide–FUS rats compared to saline–FUS‐treated (*n* = 5) animals was seen 1 week post–FUS treatment (Figure 11). Loperamide and FUS treatment had no effect on l‐selectin and CNTF expression in the proximal and distal colon (*P >* 0.050;Figure A7). Table 2 provides summary inflammatory marker data for rats treated with saline and loperamide with and without FUS treatment on day 25.

**TABLE 2:** Summary of inflammatory markers for rats treated with saline and loperamide with and without FUS treatment on day 25.

	Proximal colon				Distal colon			
	Effect of loperamide		Effect of FUS		Effect of loperamide		Effect of FUS	
Inflammatory markers	−FUS	+FUS	Within saline groups	Within loperamide groups	−FUS	+FUS	Within saline groups	Within loperamide groups
**IL‐2**	—	—	—	—	↑	—	—	↑
**IL‐10**	—	—	—	—	—	—	—	—
**IL‐3**	—	—	—	—	—	—	—	—
**IL‐17**	—	—	—	—	↑	—	—	↓
**IL‐4**	—	—	—	—	↑	—	—	↓
**IL‐1ra**	—	—	—	—	↑	↑	—	—
**IL‐6**	—	—	—	—	↑	—	—	—
**IFN‐γ**	—	—	—	—	—	—	—	—
**IL‐1α**	—	—	—	—	↑	—	—	↓
**IL‐1β**	—	—	—	—	↑	—	—	↓
**IL‐13**	—	—	—	—	—	—	—	↓
**TNF‐α**	—	—	—	—	—	—	—	—
**GM‐CSF**	—	—	—	—	↑	—	—	—
**CXC3CL‐1**	—	—	—	—	—	—	—	—
**CXCL‐10**	—	—	—	—	↑	—	—	—
**CXCL‐7**	—	—	—	—	—	—	—	—
**CCL‐3**	—	—	—	—	↑	—	—	↓
**CXCL‐3**	↑	—	—	↓	↑	—	—	—
**CXCL‐5**	—	—	—	—	↑	—	—	—
**CCL‐5**	—	—	—	—	—	—	—	—
**CXCL‐1**	—	—	—	—	—	—	—	—
**CXCL‐2**	—	—	—	—	↑	—	—	↓
**CXCL‐9**	—	—	—	—	↑	—	—	—
**CCL‐20**	—	↑	—	—	↑	—	—	—
**l‐Selectin**	—	—	—	—	—	—	—	—
**CNTF**	—	—	—	—	—	—	—	—
**TIMP‐1**	—	—	—	—	—	↑	—	—
**VEGF**	—	—	—	—	—	↑	—	↑
**ICAM‐1**	—	—	—	—	↑	—	—	—

An arrow indicates significance, and the direction denotes an increase or decrease based on saline compared to loperamide and without FUS compared to with FUS treatment.

## 4 DISCUSSION

We observed that FUS‐treated groups had increased faecal pellet output and reduced dry weight per pellet both during and after FUS treatment. These animals also had improved weight gain when compared to loperamide‐treated controls. Cytokine profiling revealed reduced levels of pro‐inflammatory cytokines, chemokines and acute phase proteins in proximal and distal colon tissue in FUS‐treated groups, but no histological differences in epithelial damage and immune cell infiltration between groups. Taken together, our findings posit that the mitigation of constipation in the loperamide rat model may be accomplished with non‐invasive peripheral FUS on a specific arm of the peripheral nervous system with select changes in cytokine expression to improve GI dysmotility.

Loperamide is widely used to establish animal constipation (Ayari et al., 2025; Kim et al., 2018; Liu & Zhi, 2021; Wintola et al., 2010; Zhan et al., 2021), and is a reliable model of spastic constipation (Lim et al., 2019). In contrast to functional constipation that occurs without any identifiable cause (Allen et al., 2026), we observed the effects of FUS on opioid‐induced constipation. Loperamide works as an opioid‐receptor agonist by inhibiting intestinal motility (Li et al., 2025) and increasing colonic contractions (Lim et al., 2019), but an overabundance of the drug can result in cardiotoxicity as early as 7 days (Olofinsan et al., 2019). As a result, we developed our paradigm with rest days in which the rats did not receive loperamide administration to help prevent adverse non‐constipation side effects. While this may affect the fluctuations of constipation, we ensured that the time between loperamide injection and FUS treatment was consistent, minimizing these fluctuations in our data. In future studies, a model of constipation can be employed based on diet or environmental factors to examine the effects of FUS on chronic constipation with our current study positing FUS having a therapeutic effect on spastic constipation.

Previous findings demonstrated that loperamide‐induced constipation reduced the number of faecal pellets produced by the animal model (Ju et al., 2020; Kim et al., 2018; Liu & Zhi, 2021), which was supported by our study. While assessing water content in faeces may provide a direct measurement of colonic transit time and body hydration levels, rat faeces lose moisture rapidly with wet weight data unreliable with our intermittent monitoring during the longitudinal experimental design in home cages. In future studies, we may examine water content in faeces, but we assessed dry pellet weight as others did with loperamide‐induced models (Ju et al., 2020; Kim et al., 2018; Liu & Zhi, 2021). In support of the potential therapeutic effects of FUS, we found that loperamide‐injected FUS‐treated animals had an increased number of faecal pellets compared to loperamide‐injected animals without FUS treatment. Further, given that the AUC analysis was statistically significant during both FUS treatment periods and during recovery, our findings may indicate there are short‐ and long‐term FUS effects. Notably, FUS treatment significantly improved weight recovery in loperamide–FUS‐treated rats compared to loperamide alone, during both FUS treatment and the recovery phase, further demonstrating the potential acute and long‐term benefits. Even a week after FUS treatment had ended, some constipation symptoms were still alleviated. Therefore, these findings suggest that FUS may mitigate metabolic disruptions caused by prolonged constipation, potentially through its anti‐inflammatory effects and restoration of normal gut motility (Cotero et al., 2020; Wang et al., 2023; Zhang et al., 2021).

Previous research using loperamide‐induced constipation demonstrated increased cytokine expression (i.e., IL‐1β and TNF‐α) in the colon, along with elevated levels of inflammatory mediators such as glial cell line‐derived neurotrophic factor, transient receptor potential vanilloid 1 and nitric oxide synthase (Ju et al., 2020; Liu & Zhi, 2021). Additionally, studies have reported reduced ACh levels in constipated rats (Liu & Zhi, 2021). Collectively, these findings suggest that targeting inflammation by reducing pro‐inflammatory cytokines and enhancing ACh levels can improve motility and effectively alleviate constipation by targeting the neuroimmune interaction within the VN (Ju et al., 2020; Kim et al., 2018; Wintola et al., 2010). In this study, we have demonstrated the potential of FUS treatment and both metabolic and physical benefits on a loperamide‐induced constipation model (Ju et al., 2020; Kim et al., 2018; Wintola et al., 2010). We found that non‐invasive FUS is a novel therapeutic to alleviate loperamide‐induced constipation through increased faecal pellet output, improved metabolic outcomes and inflammatory mediators 1 week post‐treatment. This innovative approach holds promise as a non‐invasive treatment option for managing constipation and related inflammatory conditions.

Loperamide had minimal impact on food and water consumption in our animals, albeit these changes are inconsistently reported by others. Liu and Zhi (2021) did not find significant effects on food consumption or body weight change but noted the loperamide‐treated group drank more than control groups (Liu & Zhi, 2021). In contrast, Ayari et al. (2025) found the inverse with loperamide‐treated rats eating and weighing less than control rats but finding no significant effect for water consumption (Ayari et al., 2025). Though we found significant differences between loperamide and saline groups for body weight change, no change was found between groups for food or water consumption. Interestingly, we had comparable results to both studies, as Ayari et al. (2025) found loperamide animals had at least a 5% decrease in body weight, compared to our average of about 10% after day 9, and Liu and Zhi (2021) found that loperamide‐treated animals drank about 13 mL more than control groups, a slightly smaller value to our significant difference between groups on day 17. We followed a comparable loperamide‐injection paradigm as Liu and Zhi (2021), where animals received two doses a day for 3 days, compared to Ayari et al. (2025), who did one dose for 7 days (Ayari et al., 2025; Liu & Zhi, 2021). With that said, we slightly revised this protocol by separating the two loperamide injection paradigms with a brief 2‐day pause. This may explain why the only day when food consumption increased was after one of the first injection days, with animals having more time to adjust their eating and drinking habits to accommodate the high double daily dose of loperamide. Therefore, whether rats have increased food and/or water consumption that affects body weight changes could rely on the specific dosing and timeline of the loperamide injection paradigm.

The increase of chemokines in the FUS treatment group for saline‐treated rats posits a localized inflammatory response to the ultrasound (Ju et al., 2020). CCL‐3 is important for immune cell recruitment for repair and pathogen clearance (Bhavsar et al., 2015) and angiogenesis via CXCL‐7 (Wu et al., 2022). An increase of distal colon CXCL‐7 and CCL‐3 in saline groups receiving FUS may indicate the underlying processes of the body on neuroimmune modulation. Meanwhile, FUS‐treated loperamide‐injected animals had significantly decreased levels of both CXCL‐7 and CCL‐3 on day 18 compared to loperamide‐injected animals without FUS treatment, which may underscore an alternative route for therapy without engaging the body's natural response to elevate these cytokines. It has also been found that IL‐6 is a primary inflammatory protein that may mediate inflammation, so the increase generated by FUS treatment may stimulate the cascade and can ultimately lead to the resolution of inflammation (Chen et al., 2018). Another crucial protein, ICAM‐1, plays a critical role in mediating interactions between monocytes and endothelial cells, producing CCL‐3 (Lukacs et al., 1994). We found an increase in ICAM‐1 expression observed in the saline groups treated with FUS, indicating it also has a primary role in the inflammatory cascade. Overall, the presence or absence of inflammatory mediators observed in loperamide‐treated and FUS‐treated rats, compared to the corresponding control group, highlight the selective anti‐inflammatory effects of FUS in the context of loperamide‐induced inflammation.

The observed differences between the proximal and distal colon at days 18 and 25 demonstrate region‐specific responses to loperamide and FUS treatment. On day 18, the distal colon exhibited more inflammatory responses than the proximal colon, as many of the distal colon proteins were significantly different between groups, compared to no found differences in the proximal colon. This was demonstrated by significant increases in IL‐2, IL‐4, IL‐17, CXCL‐7 and CCL‐3 for the loperamide‐treated animals without FUS compared to both saline‐treated and loperamide‐and‐FUS‐treated animals. Prior studies suggest that variations in immune cell composition and neural innervation across the GI tract may contribute to differences in inflammatory resolution (Hickey et al., 2023), and we posit that the proximal colon, with its higher abundance of immune mediators, may experience faster resolution times (Hickey et al., 2023; Zhang et al., 2021), hence less immune response effects in the proximal colon compared to the distal colon on day 25 (Table 2). Further, spatial differences in the proximal and distal colon, including their innervation systems (Morales‐Soto & Smith‐Edwards, 2025), may, in part, explain these findings. While some colitis models have found cytokine differences between the proximal and distal colon (Alex et al., 2009), this area is understudied for constipation models, and our research begins to tease apart the inflammatory mediator differences for each colon.

We found that the cytokines and chemokines most impacted by FUS were IL‐1β and IL‐6 and CXCL‐7 and CCL‐3, respectively. Notably, the latter are platelet‐derived (Flad & Brandt, 2010) and the former mediate or trigger platelet‐derived growth (Lindemann et al., 2001; Roth et al., 1995). In contrast, CXCL‐9 was not impacted by FUS treatment at day 18 in the proximal colon or day 25 in both colons and is produced by immune and structural cells (Tokunaga et al., 2018). Taken together with that others reported high‐intensity FUS can activate platelets and stimulate primary haemostasis (Poliachik et al., 2001), it is plausible FUS treatment may be most effective on platelet‐derived inflammatory mediators and we speculate that this could serve as a long‐term therapeutic by averting negative impact on healthy cell‐to‐cell signalling in light of structural cells being less affected.

Importantly, histological analyses of the colon did not reveal any epithelial damage or immune cell infiltration, so inflammatory mediator differences can be analysed without regard for potential side effects. Further, chemokines are widely recognized in the initiation and propagation of inflammation (Wu et al., 2022). However, the precise sequence of immune responses during the resolution phase remains incompletely understood. We observed sustained expression of CXCL‐3 throughout the recovery period in both the proximal and the distal colon. Given its critical role in maintaining gut homeostasis, the prolonged presence of CXCL‐3 in the colon during recovery is particularly intriguing with its potential functions in the resolution of inflammation (Cheng et al., 2023) and restoration of gut health in prolonged use of loperamide (Whittaker & Newman, 2021). In line with these findings, at day 25, increased VEGF expression in the distal colon of loperamide groups treated with FUS may suggest the role of FUS in promoting prolonged angiogenesis and recruitment of immune cells beyond the inflammatory phase (Melincovici et al., 2018). Additionally, constipation is driven by low‐grade gut inflammation, and pro‐inflammatory cytokines can impair motility via the ENS when released after the intestinal lining is compromised (Docsa et al., 2022). Our data indirectly supports this notion with an increase in these specific cytokines (i.e., TNF‐α, IL‐1β, IL‐6). While the scope of the current study was to provide initial data and proof‐of‐concept that FUS can elicit anti‐inflammatory signals via chemokines and cytokines, next steps would include interrogating the relationship between the neural and immune systems with the evaluation of the effects of FUS on enteric neurons.

For future studies, it would be informative to study FUS efficacy on constipation using female rats given our study employed only male rats. Moreover, investigating this with diet‐induced or spontaneous genetic models may provide further insight into the translational relevance of FUS. Lastly, the potential involvement of the cholinergic anti‐inflammatory pathway for the underlying effects of FUS on the celiac plexus would need to be teased apart. Constipation is associated with reduced activity of noradrenaline and ACh in the myenteric and submucosal plexus due to decreased activation of μ‐opioid receptors, ultimately disrupting motility and immune signalling within the GI tract (Kim et al., 2018; Sobczak et al., 2014; Wang et al., 2023). Investigating the effects of FUS on ACh and noradrenaline levels in these enteric plexuses may lend insights into whether modulation of a neuro‐immune pathway is an effective strategy for reducing gut inflammation to improve GI motility and constipation (Wang et al., 2023).

Overall, this study highlights the therapeutic potential of FUS in treating intestinal inflammation by providing evidence that FUS treatment can reduce the metabolic, inflammatory and immune disturbances associated with loperamide‐induced constipation in rats.

## AUTHOR CONTRIBUTIONS

Data were collected at Albany Medical College in the Department of Neuroscience & Experimental Therapeutics. Dia Shah, Kainat Akhtar, Eric Molho, and Damian Shin conceptualized or designed the study. Tissue and data collection or interpretation of the data for the work were performed by Dia Shah, Kainat Akhtar, Dev Dwivedi, Amal Siddiqui, Griffin Haggas, Nicole Forman, Yifan Kao, John Chen, Tatiana Gervase, Julia Brzac, Eric Molho, and Damian Shin. Dia Shah, Kainat Akhtar, Dev Dwivedi, Amal Siddiqui, Griffin Haggas, Nicole Forman, Yifan Kao, John Chen, Tatiana Gervase, Julia Brzac, Eric Molho, and Damian Shin participated in manuscript writing or critical for intellectual content. Manuscript review was conducted by Dia Shah, Kainat Akhtar, Dev Dwivedi, Amal Siddiqui, Griffin Haggas, Nicole Forman, Yifan Kao, John Chen, Tatiana Gervase, Julia Brzac, Eric Molho, and Damian Shin. Finally, study supervision was conducted by Dia Shah, Kainat Akhtar, and Eric Molho with study oversight by Damian Shin. The corresponding author had full access to all the data in the study and takes responsibility for the integrity of the data and the accuracy of the data analysis. All authors have read and approved the final version of this manuscript and agree to be accountable for all aspects of the work in ensuring that questions related to the accuracy or integrity of any part of the work are appropriately investigated and resolved. All persons designated as authors qualify for authorship, and all those who qualify for authorship are listed.

## CONFLICT OF INTEREST

Eric Molho consults for Acadia Pharmaceuticals and receives clinical trial grant support from Cure Huntington Disease Initiative/Huntington Study Group, Cerevel Therapeutics and Parkinson Study Group/NIH and educational fellowship grant support from Abbott, AbbVie, Amneal, Boston Scientific, Medtronic and Merz North America.

## GENERATIVE AI STATEMENT

No generative AI tools were used in the preparation of this manuscript.

## Data Availability

The data that support the findings of this study will be made available from the corresponding author upon request.
